# Why we need a small data paradigm

**DOI:** 10.1186/s12916-019-1366-x

**Published:** 2019-07-17

**Authors:** Eric B. Hekler, Predrag Klasnja, Guillaume Chevance, Natalie M. Golaszewski, Dana Lewis, Ida Sim

**Affiliations:** 1 Center for Wireless & Population Health Systems, Department of Family Medicine and Public Health, Design Lab and Qualcomm Institute Faculty Member, UC San Diego, 9500 Gilman Ave, San Diego, CA 92093 USA; 2 School of Information, University of Michigan, Ann Arbor, MI USA; 3 OpenAPS, Seattle, Washington USA; 4 School of Medicine, UC San Francisco, San Francisco, CA USA

**Keywords:** Precision medicine, Personalized medicine, Precision health, Small data, Artificial intelligence, Data science

## Abstract

**Background:**

There is great interest in and excitement about the concept of personalized or precision medicine and, in particular, advancing this vision via various ‘big data’ efforts. While these methods are necessary, they are insufficient to achieve the full personalized medicine promise. A rigorous, complementary ‘small data’ paradigm that can function both autonomously from and in collaboration with big data is also needed. By ‘small data’ we build on Estrin’s formulation and refer to the rigorous use of data by and for a specific N-of-1 unit (i.e., a single person, clinic, hospital, healthcare system, community, city, etc.) to facilitate improved individual-level description, prediction and, ultimately, control for that specific unit.

**Main body:**

The purpose of this piece is to articulate why a small data paradigm is needed and is valuable in itself, and to provide initial directions for future work that can advance study designs and data analytic techniques for a small data approach to precision health. Scientifically, the central value of a small data approach is that it can uniquely manage complex, dynamic, multi-causal, idiosyncratically manifesting phenomena, such as chronic diseases, in comparison to big data. Beyond this, a small data approach better aligns the goals of science and practice, which can result in more rapid agile learning with less data. There is also, feasibly, a unique pathway towards transportable knowledge from a small data approach, which is complementary to a big data approach. Future work should (1) further refine appropriate methods for a small data approach; (2) advance strategies for better integrating a small data approach into real-world practices; and (3) advance ways of actively integrating the strengths and limitations from both small and big data approaches into a unified scientific knowledge base that is linked via a robust science of causality.

**Conclusion:**

Small data is valuable in its own right. That said, small and big data paradigms can and should be combined via a foundational science of causality. With these approaches combined, the vision of precision health can be achieved.

## Background

A variety of global initiatives are advancing ways of providing more personalized and precise care to individuals. These initiatives go under various monikers, such as ‘precision medicine’ in the US and ‘personalised medicine’ in the UK, but it is herein referred to as precision health. The general focus of precision health is on prevention and treatment strategies that take individual differences into account [1]. These efforts are being advanced in several nations, including the All of Us Research Initiative in the US and the 100,000 Genomes Project in the UK, with a current focus on identification of actionable genetic mutations that predict response to cancer treatment.

Precision health is both old and new. It is old in that it aligns with evidence-based practice [2], which emphasizes the use of evidence and clinical expertise to make clinical decisions that take individuals’ physiology, condition, and circumstances into account. Such matching of treatment to individual differences takes many forms; indeed, blood type is a classic example of matching interventions (in this case blood transfusion) to individual differences. Another example is adjusting the dosage of a drug, such as anti-retroviral treatments, based on well-measured, dynamic clinical markers (e.g., white blood cell count), using clearly specified if/then logic to drive adaptive dosing. In the realm of public health, support individuation has taken the form of matching adaptive and ‘tailored’ support through coaching for complex issues such as preventing and treating obesity.

The new element in precision health arises from new data, informatics tools, and data analytic technologies [3–5], which promise to advance individualization. Many new data types (e.g., whole genome sequencing or wearable device, microbiome, or environmental exposure data) offer unique insights into health phenomena. Robust informatics infrastructures are being developed to support the rigorous and efficient collection, storage, retrieval, and organization of data. Finally, artificial intelligence, machine learning, data science analytics, and ‘-omics’ sciences (e.g., genomics, metabolomics, microbiomics) offer new possibilities for gleaning insights from data that go well beyond classic evidence-based practice. We label the majority of currently used data analytic techniques as ‘big data analytics’ since researchers commonly conduct these data analyses with new data types via robust informatics infrastructures, with the insights sought often aimed towards helping other individuals, beyond those for whom the data were collected.

While insights from big data analytics are essential, they are insufficient. A rigorous ‘small data’ paradigm that functions autonomously and collaboratively with big data analytics is also needed. By ‘small data’ we build on Estrin’s formulation [6] and refer to the rigorous use of data collected to advance the goals of the specific N-of-1 unit for whom the data are about (i.e., a single person, clinic, hospital, healthcare system, community, city, etc.). The goal of a small data approach is to achieve improved individual-level description, prediction and, ultimately, control for that specific unit. As part of this, the unit itself plays a role in defining the objectives of data analysis. In contrast, a ‘big data’ approach refers to the use of data collected from one set of individuals with the goal of improved description and prediction of a phenomenon for other individuals, not necessarily those from whom the data were collected. This is typically done by some other entity, such as a researcher, company, or health insurance group, with the individuals whose data formed the datasets often not involved in defining data use objectives. As such, most health science research methods, such as epidemiology and clinical trials, including randomized controlled trials, fit into a big data approach, coupled with the many current uses of artificial intelligence, machine learning, and other approaches more commonly linked with ‘big data’. While we are using the word ‘small’ as a counter to ‘big’, these data are ‘small’ only in the sense that the data are collected from and are being used for a single unit. Indeed, an N-of-1 unit could have a very large dataset in terms of data types (e.g., the various -omics data) and length of time series data (e.g., years).

The purpose of this piece is to articulate why a small data paradigm is needed and valuable in itself, and to provide initial directions for future work that can advance study designs and data analytic techniques for a small data approach to precision health in a complementary and explicitly not subservient way to a big data approach.

### Why we need a small data paradigm

#### Scientific reason

At the heart of precision health is the notion of individualizing treatment based on the specifics of a single unit. Matching the right intervention to the right individual at the right time, in context, is contingent upon the inherent complexity of a phenomenon. On the simple end are problems like matching blood transfusions to blood types, which is relatively straightforward since the problem is (1) not dynamic (i.e., blood type does not change), (2) there is only one key cause (i.e., heredity), and (3) the mechanism is well understood and easily measurable to support clear classifications (e.g., type A, B, O, AB, +/−). A more complex problem is supporting adaptive dosing, such as anti-retroviral care, where the phenomenon is (1) dynamic (i.e., dosage is contingent upon changing white blood count) and (2) multi-causal, as a wide range of factors, beyond just the person’s disease state, influence white blood count. Nevertheless, often, such problems can be simplified into if/then adaptation rules because, like blood type, the mechanism is well-understood and characterized with appropriately validated measures. For problems in this class (i.e., low to moderate complexity), the big data approach to precision health will be very valuable.

However, there are highly complex health problems whose characteristics are poorly matched to using a big data approach alone. A good example of such problems is obesity prevention and treatment. As illustrated elsewhere [7], obesity is highly complex since it is dynamic and multi-causal, and the mechanisms – even seemingly universal ones such as energy balance – manifest idiosyncratically. For example, it is well known that eating less facilitates weight loss. However, each person ‘eats less’ or struggles with eating less differently, based on food preferences, cultural practices, food access, time of day, learning history, etc. The level of calorie restriction required also varies, thus suggesting physiological differences. Individualizing prevention and treatment likely require that those idiosyncrasies be accounted for. Modest successes, particularly for achieving robust weight loss maintenance [8, 9], suggest room for improvement for supporting individuals. As most major health issues today are chronic as opposed to acute [10], in all likelihood, the level of complexity of the problems we seek to address will increasingly be closer to that of obesity than of blood type.

If the problems we face are more akin to obesity than to blood type, then the big data approach alone will be insufficient since the more dynamic, multi-causal, and idiosyncratically manifesting a problem is, the harder it will be to obtain the appropriate data types of meaningful causal factors at the appropriate temporal density from a large enough number of units. Data analytics that are based, in part, on identifying clusters and patterns across people will experience exponential growth of complexity of the modeling space, and thus require huge samples with long time series. Nevertheless, increasingly large datasets are becoming available. Thus, big data will play an important role, such as modeling variations in comorbidities across units.

Even with the large datasets available, the big data approach requires a great deal of knowledge about a phenomenon to ensure the right data types are included. For example, race is commonly measured, partially because it is relatively easy to measure via self-report and uses ‘standardized’ categories. Prior work is challenging assumptions about the meaning of this variable, particularly an implicit assumption that race is a biological as opposed to a socially constructed concept. ‘Race’ is largely contingent upon the cultural context for which an individual exists within [11]. It is quite plausible that the categories of race create more noise than signal when used, particularly if they are treated as biological, immutable realities, which could propagate inequities from the research conducted [12]. This issue will only magnify when data are aggregated across individuals. While we recognize this issue with race, it is quite plausible that similar hidden misclassifications exist, thus creating a high risk of inappropriate conclusions from big data. A central task, then, even when the goal is to use big data approaches, is to advance ways of gathering complementary prior knowledge to understand and analyze a complex phenomenon. This has classically occurred through clinical expertise and qualitative methods and, as justified herein, could be further supported with a small data approach.

Even if this colossally complex issue of obtaining the right data types at sufficient temporal density from a large enough sample based on robust prior knowledge were solved, if the mechanism is known to manifest idiosyncratically (see [13] for many concrete examples), then big data will become not just insufficient but, potentially, problematic as it may wash out or ignore meaningful individual differences. For example, the behavioral science version of reinforcement learning (i.e., increasing future behaviors via giving rewards, like giving a dog food after sitting) is one of the most well understood drivers of behavior across organisms [14, 15]. While the mechanism is universal, it manifests idiosyncratically [14, 15]. Think, for example, of the pickiness of children. One child might find strawberries to be a reward whereas another child might find them to be aversive. Learning histories and individual preferences combine to create tremendous variability in how different people respond [13] to both specific elements in the environment (e.g., strawberries) as well as classes of those elements (e.g., dessert). These concrete details of mechanism manifestation will be averaged out in aggregated analyses, yet it is precisely at that level of concreteness that treatments have to be individualized [14–16]. Because of its focus on advancing goals of an N-of-1 unit and inclusion of that N-of-1 unit in the process, a small data approach has unique capabilities for issues that manifest idiosyncratically and, thus, are important for advancing precision health.

A small data approach uses different strategies to understand dynamic, multi-causal, and idiosyncratically manifesting phenomena, which can help to make these complexities more manageable. Within a big data paradigm, there is an implicit requirement that all plausibly meaningful variation is included in the dataset at a large enough scale to enable meaningful clusters and relationships in aggregate to be gleaned. Without this, what has been called ‘the black swan effect’ [17], can occur, whereby a rare phenomenon not in a dataset is not deemed possible and, thus, not part of the modeling efforts. Using a small data approach, there is an incentive for people for whom the data are about to think carefully through insights collected from the data and, thus, to engage in gathering the right data types at sufficient temporal density to enable them to gather actionable insights for improved prediction and control for themselves. Further, a great deal of causal factors can be ruled out based on attributes of the person, context, or time, with the individual unit playing an important role in ruling out these possibilities (e.g., “I never eat those types of food; I’m not ever exposed to those environmental issues”). An individual understands their own lives, contexts, and preferences, which can facilitate specifying the idiosyncratic manifestations that need to be measured. For example, an individual may know – or could quickly learn – the degree to which salty foods versus sugary foods might trigger them to over eat. Finally, as discussed in detail below, a small data approach targets helping individuals first, not transportable knowledge first, which enables insights to be gleaned from data without the higher bar of those insights being generalizable to others. 

In summary, from a scientific perspective, a small data approach has unique, complementary strategies for managing complex, dynamic, multi-causal, idiosyncratically manifesting phenomena compared to a big data approach, which could be valuable regardless of their value to big data approaches as well as for improving big data analytics.

### Practical reasons for advancing a small data approach

There are three practical arguments – a small data approach (1) uses success criteria that match the goals of individuals, clinicians, healthcare systems, and communities; (2) can facilitate more rapid agile learning from each unit; and (3) can offer a unique pathway to transportable knowledge.

#### Small data aligns activities to the success of individuals, clinicians, healthcare systems, and communities

The central defining feature of a small data approach is that data are being used by and for individual units themselves for their own purposes [6]. This means that the goals and desires of the individuals for whom the data are about are, by definition, used to partially define successful data use. There is an increasing number of technologies that fit with this goal, such as helping individuals identify which foods impact irritable bowel syndrome symptoms [18], which sleep hygiene recommendations are appropriate for them [19], determining if a particular evidence-based behavioral intervention ‘works’ for a particular person [20], or creating an individualized behavior change plan [21]. In contrast, a big data approach seeks to produce transportable knowledge first [22]. By transportable knowledge, we mean insights that are gathered from a group of observed units applicable to a different group of units and using it instead of generalizability based on possible confusion with the term [23].^1^ In a big data paradigm, the people who benefit are other individuals, not the individuals for whom the data are about. Small data, by definition, aligns the goals of data analytics and individuals.

Turning to clinicians, healthcare systems, and population health, the central goal of evidence-based medicine is a practical one – to help specific units (e.g., individuals, families, physicians) get better. Yet, while success for clinical care is tied to improvement in individual units, success in evidence-based medicine research – first and foremost, randomized controlled trials – is fundamentally about average improvements across abstract, artificially created groups. A small data approach emphasizes the same success criteria as clinical practice, thus better aligning science and practice towards a common goal. This same alignment of data analytics and practice also holds true for other units, including a single healthcare system, city, region, or other core population [24]. Based on this, a small data approach may not only be valuable for individuals and clinicians, but also for advancing the vision of a learning healthcare system [25] and population health.

Small data might not only be valuable scientifically for big data (to bring in prior knowledge to support appropriate categorization and articulation of measurement approaches) but also be practically valuable for big data efforts. Large scale projects, such as All of Us in the US, require sufficient data types (e.g., whole genome sequencing, wearable device data, microbiome data, environmental exposures data, etc.) at appropriate temporal density (e.g., daily for some concepts) from a large number of people. This requires a great deal of participant engagement. Based on the focus of small data, it is more likely that more people will engage with data collection as they receive direct benefit, thus helping to establish the pre-conditions for engagement with the types of studies needed to use big data analytics.

#### Small data can facilitate more rapid agile learning from each unit

**Fig. 1:**
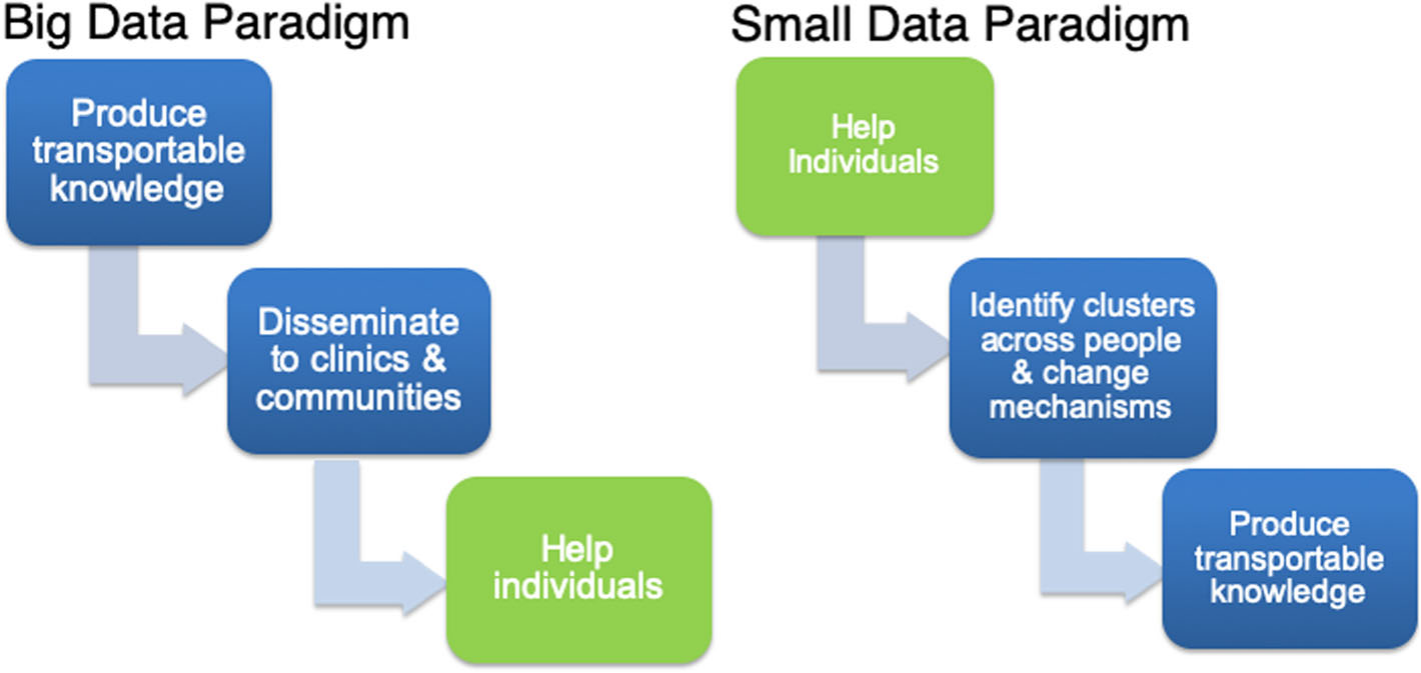
Small versus big data paradigm pathways to help individuals and transportable knowledge

As discussed elsewhere [26], it takes a long time for transportable knowledge to be disseminated and implemented in clinics or communities of practice towards helping individuals (Fig. 1). A small data paradigm, with its use of success criteria matched to the individual unit, can very likely learn more rapidly; this basic point was articulated well by Staddon [15]. If a well-specified prediction is made and it did not come to pass within a specific unit via replications within that individual, the prediction was wrong for that unit; there is no need for replication with others. Instead, the next step is to ask why the prediction did not pan out for that unit, including the quality of measurement or methods, understanding of the phenomenon, specified success criteria, study implementation, etc. When description, prediction, or control does not occur for an N-of-1 unit, that is sufficient to trigger reflection and learning. Robust individual predictions are, arguably, how key advances in physics have occurred, for example, Einstein’s very precise prediction about how light would bend around objects of great mass such as the sun. Only one observation was needed to suggest Einstein’s predictions better aligned with reality compared to Newton’s. As we articulate within agile science [16, 27], carefully defined proximal outcomes, including those that can be defined for a single unit, can greatly speed the pace of learning with less resources.

#### Small data offers a unique pathway to transportable knowledge that could be grounded in clinical practice

There is a plausible way to produce transportable knowledge from small data, as illustrated in Fig. 1. Specifically, after meaningful success is achieved for an individual, clusters of actionable insights, particularly about key mechanisms of change, can then occur. However, the clustering would be different from that of big data clustering as it would occur based on mechanisms and models of mechanisms that achieved meaningful success for each individual. For example, our prior work illustrates how system identification [28] (an approach used in control systems engineering, which could be thought of as an N-of-1 hypothesis-driven approach) can be used to identify individualized predictive models for each person related to their physical activity [27, 29]. In this work, some individuals’ steps were best predicted by day of the week whereas, for others, some other variable(s), such as stress or busyness, or a combination of factors, were most predictive. If a big data approach of aggregation across individuals had been used, an inappropriate tailoring variable would have been selected for 75% of participants, thus establishing the importance of small data methods [27, 29]. These different models for each unit (see our prior papers [29, 30]) could be used as the starting point for clustering individuals based on the models and not individual variables. Such clustering of models corresponds to the second step in the above visualization and, thus, offers a pathway to transportable knowledge. This knowledge could then be vigorously vetted by clearly specifying hypotheses of transportability and then using the emerging science of causality to vet the hypotheses (third step on right side of Fig. 1) [22].

### Limitations of a small data approach

While we see great value in a small data approach, just like big data, there are limitations. First and foremost is concern that this approach will not be available for many individual units and, instead, only possible for individuals with sufficient skill and understanding of data and data analytics and, by extension, groups such as healthcare organizations, cities, or larger, that have the technical expertise to do this work. Further, the goal of small data being used by and for the individual for whom the data are about is particularly challenging in this respect. Without careful thought and reflection, this point could be a pathway towards propagating or furthering existing inequities, as those with means can continue to learn from data whereas those without will not. This is a critical issue that requires careful thought and reflection on when to use small data as well as building capacity to facilitate equitable small data use.

With that said, the work of Lewis illustrates a concrete example of a small group of individuals using their data for their own purposes and how their work can function in a complementary fashion to big data efforts and positively influence them. Specifically, Lewis and collaborators developed components for a DIY artificial pancreas system and licensed it to be available through open source (www.openaps.org) for individuals as well as any interested researchers or commercial organizations. Their work in the OpenAPS community has had a clear impact on the type 1 diabetes research community as well as on corresponding big data efforts by influencing the pace of FDA approval for commercial artificial pancreas systems, impacting the design of new systems, and playing active roles in both advising and working within research and commercialization efforts [31]. As this example illustrates, these limitations can be overcome to help more individuals when small and big data efforts work synchronously.

Beyond this, there is also concern for the potential biases that can be brought into the scientific process due to the ‘subjective’ nature of individuals and their beliefs. Classically, the approach in science is to strive for an ‘objective’ view on reality to guide decision-making. A counter argument for this view was seeded in the work of Michael Polanyi in the 1950s. As Polanyi stated in his book, Personal Knowledge, “*… complete objectivity as usually attributed to the exact sciences is a delusion and is in fact a false ideal*” [32]. While Polanyi articulates a variety of reasons for this, some key points include that, (1) since scientists are humans, they will always bring their personal knowledge into their assessment of a situation, thus establishing the need to understand how that personal knowledge may influence conclusions drawn from evidence and (2) perhaps more importantly, a person’s personal knowledge, particularly the tacit knowledge they hold, which they cannot necessarily convey using language (think the skills of engaging in a craft such as being an artist, mechanic, surgeon, or the like), plays an essential role in guiding a person’s decision-making. This tacit knowledge is valuable in itself and should be acknowledged even if not conveyed via language alone. This philosophical stance is increasingly being supported by insights obtained from neuroscience [13, 33]. Based on this, a small data approach may be a valuable way to incorporate the personal and tacit knowledge of individuals who experience a phenomenon into scientific discourse [34].

Finally, there are practical issues such as the difficulties that often manifest when a small data effort gets started and the need for sufficiently long time series datasets to collect insights from a small data approach. One way to conceptualize the complementarity of a big versus small data approach is that big data efforts are excellent for providing insights for a ‘warm start’ understanding of what might be going on by establishing plausible variables to measure and potential actions that one could take. In contrast, a small data approach is useful for moving beyond a warm start towards an increasingly more individualized understanding that is matched to that unit. Thus, the long history of health sciences was a very important pre-condition to advancing a small data paradigm. As illustrated in other work [35], these approaches can be quite complementary and, based on the fact that a small data approach is less common, it is time to further refine and advance these methods.

### Future work

While this paper articulates the need for a small data paradigm in precision health, future work is needed to articulate how to operationalize this approach. Key areas of future work include (1) specifying a structure for understanding the rigor versus practicality tradeoff of small data methods; (2) integrating a small data approach into real-world practices, including for individuals themselves, clinicians, healthcare systems, and communities; and (3) articulating a science that actively integrates the strengths and limitations from both small and big data approaches.

**Fig. 2:**
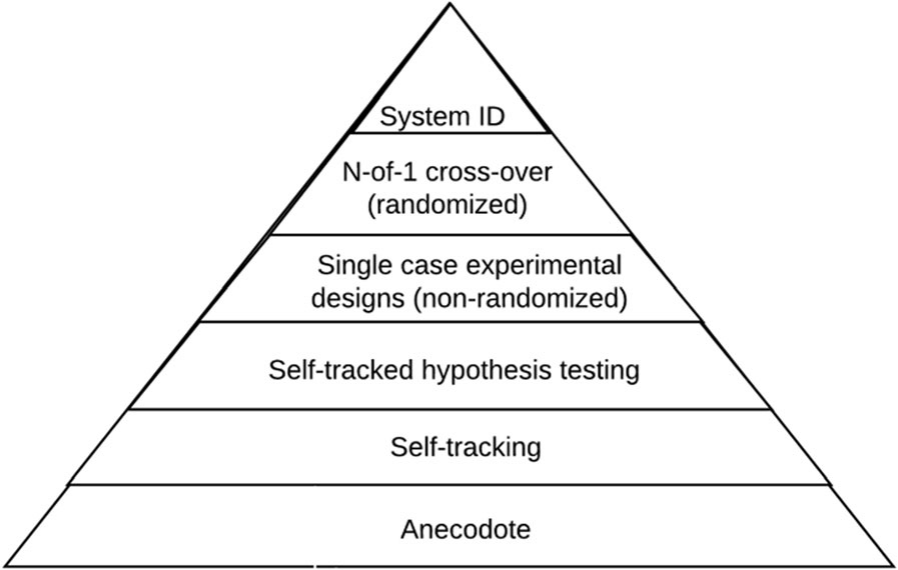
Small data hypothesis-driven pyramid

One way we situate small data methods is via the small data hypothesis-driven pyramid (Fig. 2, [36]), which highlights a way of thinking about methods from across medicine (N-of-1 cross-over designs [37–39]), behavioral science (i.e., single case experimental designs [40, 41]), and control systems engineering (i.e., system identification [28]) to achieve individualized description, prediction and, ideally, control by and for the individual unit for whom the data are about. This pyramid offers a structure for thinking through the tradeoffs between the rigor of a future prediction and control for an N-of-1 unit compared to the level of practical technical specification and expertise needed. On the bottom are study approaches that are easy for many units to implement, but sacrifice rigor in terms of prediction, causal inference, and control for the N-of-1 unit. The apex of this pyramid is system identification, which is a well-described method from control systems engineering (also called automation and control or control theory), with a wide range of tutorials available for the method [28]; for a concrete example in health, see [27]. System ID is the apex, as it is focused on improved prediction for an N-of-1 unit, which can then be directly used by an adaptive intervention (called a controller within control systems engineering) to improve control towards a desired state for an N-of-1 unit [27]. Future work is needed to vet this pyramid and to advance different ways of organizing study designs and analytic techniques.

Second, future work is needed to guide individuals, clinicians, and communities in the use of data for supporting improved individual description, prediction, and control. There are important efforts into this, such as PREEMPT [42, 43], but more work is needed, particularly to balance the real-world needs with the value gathered from small data methods. As already referenced, the field of human–computer interaction is engaging in this topic and producing a wide range of tools [18, 19, 21] that fit well into the real-world needs of people, while also honoring the principles of a small data approach. Discussions on learning healthcare systems are conceptually analogous and, thus, provide a good starting point for advancing a small data approach for N-of-1 units beyond a specific person and, instead, to individual systems, including communities.

Third, a critical area of future work is to advance the understanding of ways to combine the strengths and limitations of both big and small data approaches. To do this, two critical areas are needed – (1) specifying the different success criteria implied by different study approaches and (2) advancing the emerging science of causality as a likely bridge between big and small data.

**Fig. 3:**
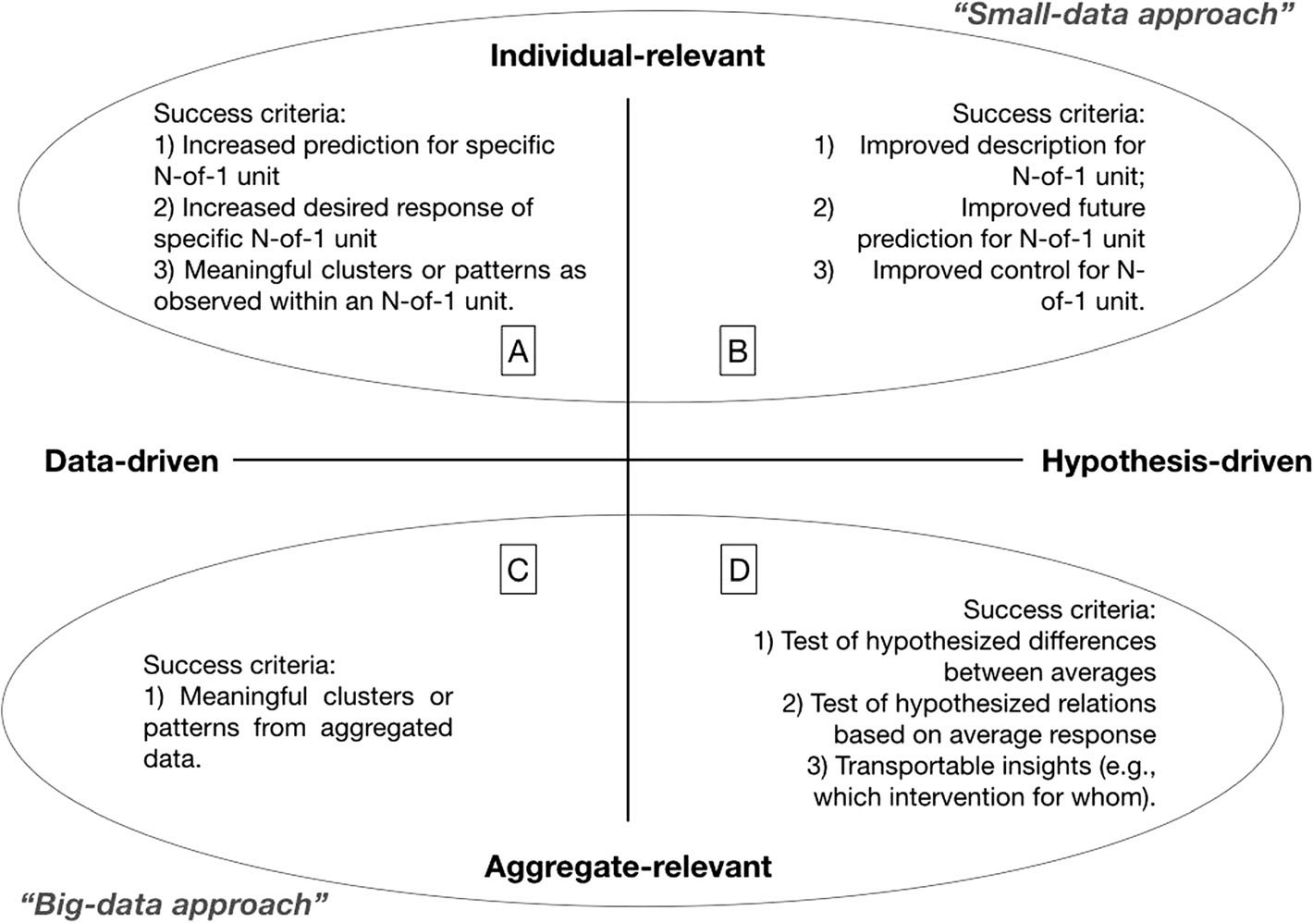
Different success criteria for big versus small data. While multiple methods can be used in each quadrant, to help illustrate, there is a rough mapping to different methods as used in different disciplines. Quadrant A includes techniques such as supervised and unsupervised machine learning, deep learning, reinforcement learning, and recommender systems, commonly used in computer science and the technology industry. Quadrant B includes techniques such as single case experimental designs, N-of-1 cross over designs, and system identification as respectively used in the social and behavioral sciences, medicine, and control systems engineering. Quadrant C includes techniques such as supervised and unsupervised machine learning and deep learning, commonly used in computer science, the technology industry, and various ‘-omics’ efforts. Quadrant D includes techniques articulated as part of the evidence-based pyramid and inferential statistics, commonly used in fields like medicine, epidemiology, public health, and psychology

As illustrated in Fig. 3, one way of organizing research studies (i.e., study design plus differing data analytic approaches) is around the success criteria of each approach. Two instructive dimensions are whether the study goals are meant to support individual units (small data) versus being more useful across an aggregation of units (big data) and if the methods are data driven versus hypothesis driven. The upper quadrants illustrate some plausible success criteria for small data analytics, with quadrant A aligning with data-driven approaches proposed by Estrin [6] and Quadrant B aligning with our small data, hypothesis-driven pyramid. Big data approaches (quadrants C and D) also include data-driven (e.g., machine learning, reinforcement learning, etc.), and hypothesis-driven (e.g., classical evidence-based pyramid in health sciences) approaches.

Building a robust understanding of a phenomenon requires the use of a diversity of methods that can be used to explore an issue [44]. When the different methods point in a common direction, consilience (i.e., a common conclusion drawn from disparate methods) can occur, thus increasing confidence in the conclusions [27, 44]. A small data approach is, arguably, a strong countervailing approach to understand health conditions that balances the limitations of big data. Similarly, big data balances the limitations of a small data approach (e.g., pure small data, not linked to the science of causality, does not produce transportable knowledge, thus setting up the need to ‘re-learn’ with each person, which would be highly inefficient when meaningful patterns exist). When small and big data approaches are combined, they offer a robust pathway for consilient knowledge of complex health phenomena.

Based on the desire for consilience, there is also a requirement for an approach that fosters triangulation of insights from disparate methods towards consilience. The emerging science of causality (e.g., [22, 45]) is very likely the foundational method for enabling effective triangulation between big and small data approaches. There are two key basic assumptions that are important from a causal perspective, namely (1) that humans (e.g., individuals, clinicians, researchers) know things that data do not know and (2) that data know things that humans do not know. The science of causality could be thought of as a rigorous way to balance those assumptions. Arguably, the movement towards big data emphasizes that data know things, with less emphasis on the idea that humans know things. There is good reason for this, as, according to Pearl and Mackenzie [22], various researchers have argued for this focus on data over human understanding; current big data efforts are, thus, a logical outgrowth of that line of thinking.

As illustrated in epidemiology (e.g., Rubin [45]) and computer science (e.g., Pearl [22]), there is increased recognition that, if the goal is not merely prediction but causal inference and, by extension, control, then a rigorous process of balancing these two assumptions is needed. There is active work advancing N-of-1 causal inference [46] and machine learning approaches [47], coupled with the more foundational causal inference already mentioned. A robust science of causality could establish a bridge across approaches and, thus, is very likely the scientific foundation for triangulating insights towards consilience to support precision health. More work is needed to advance this reality.

## Conclusion

Small data is valuable in its own right for a variety of reasons. Scientifically, a small data approach can more effectively and efficiently advance understanding of truly complex problems that are dynamic, multi-causal, and manifest idiosyncratically. Practically, small data matches success criteria of the science with those of individuals for whom the data are about, can likely speed the pace of learning, and offers a plausible unique pathway to transportable knowledge. Future work should advance ways individuals can use small data methods with their own data. This could extent to larger units such as healthcare systems and community and population health efforts. Small and big data paradigms can and should be linked via a science of causality. With these approaches combined, the vision of precision health can be achieved.

## Data Availability

Not applicable.

## Footnotes

1 In health sciences, this is often labeled ‘generalizability’. As described by Shadish et al. [23], the concept of generalization is more multifaceted than commonly considered in medicine as it can involve both whether an effect is transferable to another individual or group, what Pearl et al. [22] label transportability, as well as whether future predictions can be made for a specific N-of-1 unit. To avoid the confusion, we do not use the word generalization.
